# In Vivo Evaluation of Collagen and Chitosan Scaffold, Associated or Not with Stem Cells, in Bone Repair

**DOI:** 10.3390/jfb14070357

**Published:** 2023-07-08

**Authors:** Marcelo Rodrigues Da Cunha, Fernanda Latorre Melgaço Maia, Amilton Iatecola, Lívia Contini Massimino, Ana Maria de Guzzi Plepis, Virginia da Conceição Amaro Martins, Daniel Navarro Da Rocha, Eric Domingos Mariano, Mariáh Cationi Hirata, José Ricardo Muniz Ferreira, Marcelo Lucchesi Teixeira, Daniela Vieira Buchaim, Rogerio Leone Buchaim, Bruna Eduarda Gandra De Oliveira, André Antonio Pelegrine

**Affiliations:** 1 Department of Morphology and Pathology, Jundiaí Medical School, Jundiaí 13202-550, Brazil; marcelocunha@g.fmj.br (M.R.D.C.);; 2 Interunits Graduate Program in Bioengineering (EESC/FMRP/IQSC), University of Sao Paulo (USP), São Carlos 13566-970, Brazil; 3 Department of Implant Dentistry, Faculdade São Leopoldo Mandic, Campinas 13045-755, Brazil; 4 Sao Carlos Institute of Chemistry, University of Sao Paulo (USP), São Carlos 13566-590, Brazil; 5 R-Crio Criogenia S/A, Departamento de Bioengenharia, Campinas 13098-324, Brazil; 6 Prosthodontics Department, Faculdade São Leopoldo Mandic, Campinas 13045-755, Brazil; 7 Postgraduate Program in Structural and Functional Interactions in Rehabilitation, Postgraduate Department, University of Marilia (UNIMAR), Marília 17525-902, Brazil; 8 Medical School, University Center of Adamantina (UNIFAI), Adamantina 17800-000, Brazil; 9 Graduate Program in Anatomy of Domestic and Wild Animals, Faculty of Veterinary Medicine and Animal Science, University of São Paulo (FMVZ/USP), São Paulo 05508-270, Brazil; 10 Department of Biological Sciences, Bauru School of Dentistry (FOB/USP), University of São Paulo, Bauru 17012-901, Brazil

**Keywords:** collagen, chitosan, bone regeneration, cell culture, oral-maxillofacial trauma, mesenchymal stem cells, bone repair, scaffolds, dental pulp

## Abstract

Natural polymers are increasingly being used in tissue engineering due to their ability to mimic the extracellular matrix and to act as a scaffold for cell growth, as well as their possible combination with other osteogenic factors, such as mesenchymal stem cells (MSCs) derived from dental pulp, in an attempt to enhance bone regeneration during the healing of a bone defect. Therefore, the aim of this study was to analyze the repair of mandibular defects filled with a new collagen/chitosan scaffold, seeded or not with MSCs derived from dental pulp. Twenty-eight rats were submitted to surgery for creation of a defect in the right mandibular ramus and divided into the following groups: G1 (control group; mandibular defect with clot); G2 (defect filled with dental pulp mesenchymal stem cells—DPSCs); G3 (defect filled with collagen/chitosan scaffold); and G4 (collagen/chitosan scaffold seeded with DPSCs). The analysis of the scaffold microstructure showed a homogenous material with an adequate percentage of porosity. Macroscopic and radiological examination of the defect area after 6 weeks post-surgery revealed the absence of complete repair, as well as absence of signs of infection, which could indicate rejection of the implants. Histomorphometric analysis of the mandibular defect area showed that bone formation occurred in a centripetal fashion, starting from the borders and progressing towards the center of the defect in all groups. Lower bone formation was observed in G1 when compared to the other groups and G2 exhibited greater osteoregenerative capacity, followed by G4 and G3. In conclusion, the scaffold used showed osteoconductivity, no foreign body reaction, malleability and ease of manipulation, but did not obtain promising results for association with DPSCs.

## 1. Introduction

Oral and maxillofacial traumas are common problems in emergency services and mandibular, nasal and zygomatic fractures are the most prevalent maxillofacial traumas [1]. Considering the prevalence of these traumas, special attention is paid to avoiding unfavorable treatment outcomes such as post-traumatic deformities, infection, ankylosis, compromised facial aesthetics, laceration of soft tissues and neurovascular structures, paranasal sinus lesions, reduced upper airway function and impairment of dental occlusion and masticatory function [2].

Although several surgical methods are available for the reconstruction of craniomaxillofacial defects caused by trauma, tumor removal and skeletal abnormalities, these methods still have limitations [3,4]. For example, the use of autologous bone grafts has impairments due to morbidity in the donor area, as well as limitations in terms of the bone percentage that can be obtained [5,6,7] and the ability to restore the aesthetic characteristics of the host tissue [4]. To overcome these problems, some alloplastic biomaterials, including polymers, are being developed or improved in order to allow their more frequent use in the repair of craniofacial injuries because of their ability to mimic the functions and components of the bone matrix [4,8,9,10,11].

Polymers are macromolecules composed of repeating units of smaller molecules, called monomers, which can be of natural or synthetic origin [12]. Natural polymers used in tissue engineering include hyaluronic acid [13], collagen and chitosan. Common synthetic polymers are poly(L-lactide) (PLLA) and poly(L-lactide-co-glycolide) (PLGA) copolymers [3,14]. However, in order to be used in tissue engineering, these materials must have certain features, such as being mechanically stable, biocompatible, bioactive and biodegradable [15]. Natural polymers have been used as scaffolds in bone tissue engineering since they are capable of regulating the cell phenotype and enable cell adhesion, growth and proliferation, essential features for the formation of a new tissue [9,16,17,18,19].

Among natural polymers, collagen and chitosan matrices obtained from swine or bovine animal tissue and from crustaceans, respectively, are being used. Both materials have shown satisfactory results in experimental studies on bone regeneration, thus justifying their future application in medical and dental clinical practice [8,11,18]. Collagen is the most abundant protein of the extracellular matrix (ECM) responsible for maintaining the structural and biological integrity of various body components. Important properties of collagen include its biodegradation, water and nutrient absorption, the ability to promote cell adhesion and the absence of tumor-inducing features [20,21,22,23].

There are about 29 known types of collagen, the most common being types I, II, III and IV. Type I is the most abundant and is the main protein present in bones, skin, tendons and ligaments, corneas and blood vessels. Its basic unit is tropocollagen, which is formed by two identical chains (α1), with about 1055 amino acid residues and a different chain (α2), with about 1029 residues, creating a linear, semi-flexible molecule with 300 nm in length and 1.5 nm in diameter [24,25,26]. Collagen can be divided into fibrillar and non-fibrillar types. Fibrillars I, II and III form typical collagen fibrils, with an axial periodicity of 67 nm [27]. Since it is an important macromolecule in bones, teeth and temporomandibular joints and considering its ability to interact with mesenchymal stem cells (MSCs), collagen can be applied as a biomaterial in regenerative dentistry due to its similarity with the host ECM and its ability to elicit the cellular response necessary for tissue repair [28,29]. 

The use of collagen in scaffolds also offers a support for cell growth [30]. Collagen can be obtained from natural or synthetic resources, with natural coming from animals or herbs. Examples of animal origin are pigs, cattle, humans and marine organisms such as fish scale and skin. Less used are those from chicken, rat-tail tendons, kangaroo, duck feet, equine tendon, alligator, bird feet, sheepskin or frog. Sources of synthetic with the advantage of preventing immunological problems are the material commercially called KOD or collagen mimetic peptides [31].

Studies using bovine osteoblasts cultured on collagen scaffolds have demonstrated excellent in vitro mineralization, alkaline phosphatase production and differentiation into the osteoblastic lineage [32,33]. Furthermore, collagen can also be combined with other substances in order to improve its essential mechanical properties in load-bearing areas such as the mandible [34]. Within this context, the combination of collagen with chitosan has been shown to be a promising alternative for tissue healing purposes, given the antimicrobial and hemostatic properties of this polysaccharide; in addition, chitosan improves the structure of collagen fibers when used as a scaffold for cell growth [8].

Chitosan is found in the exoskeleton of crustaceans, mollusks and insects. Its use as a biomaterial is justified by its biocompatibility. In addition, there are no limitations in terms of quantity, it is inexpensive and can be obtained by partial deacetylation of chitin. Thus, chitosan/collagen scaffolds have been indicated for applications in tissue engineering [35]. In addition to the treatment of craniofacial defects using natural or synthetic bone grafts, MSCs also represent an interesting alternative for tissue repair because of their self-renewal capacity and multilineage differentiation. Therefore, human dental pulp-derived MSCs (DPSCs) have received increasing attention as an alternative source for cell therapy [36,37] and studies have combined these stem cells with polymeric scaffolds for craniofacial bone regeneration [38].

The function of DPSCs is to produce odontoblasts. These cells can be obtained from postnatal teeth, extracted wisdom teeth, or exfoliated deciduous teeth [39,40]. Depending on their multipotency and sensitivity to local paracrine activity, DPSCs could be used for therapeutic applications in tissue regeneration [41,42], in addition to other advantages such as immunomodulatory properties, good proliferation capacity and osteogenic differentiation [37,43,44]. Furthermore, there is an abundant source of these cells as they are obtained from discarded or removed teeth [45].

In addition to their application in tooth structure regeneration, DPSCs are also investigated for the repair of tissues other than teeth. Within this context, the use of mimetic ECM of bone tissue seeded efficiently with DPSCs could provide an ideal microenvironment for specific applications in tissue engineering aimed at bone repair [46]. In the present study, we tested a new collagen/chitosan anionic matrix with a higher collagen-to-chitosan ratio (3:1). The highest proportion of collagen occurred because it is the protein present in the bone matrix in its greatest quantity. The incorporation of chitosan occurred in the attempt to control the degradation time of the scaffold.

Collagen and chitosan are easy to obtain, but few studies correlate the effectiveness of these natural biopolymers combined with human DPSCs in the treatment of craniomaxillofacial defects, which require rapid recovery in order to morpho-physiologically restore the bone tissue. Considering these uncertainties about the use of DPSCs [31,41,42,43], and using the hypothesis that these matrices can contribute to the repair process and support for stem cells, the aim of this study was to analyze the repair of mandibular defects, filled with a new bovine collagen/chitosan scaffold, associated or not with DPSCs.

## 2. Materials and Methods

This in vivo experimental protocol aimed to analyze the repair of mandibular defects filled with a new collagen/chitosan scaffold, with or without DPSCs. The preparation and characterization of this scaffold was fully described by Massimino (2020) [47].

### 2.1. Materials

Anionic type I collagen was prepared from bovine tendon, as previously described by Horn et al. (2015) [48]. The bovine tendon was treated with an alkaline solution containing hydroxides, chlorides and sulfates (K^+^, Ca^2+^ and Na^+^) for 72 h, followed by treatment with an aqueous salt solution (K^+^, Ca^2+^ and Na^+^) for 6 h. All salts were removed with deionized water. Anionic collagen was extracted with acetic acid (pH 3.5) and then stored under refrigeration (4 °C). The concentration of anionic collagen was determined by dry mass weighing (*n* = 3) and adjusted to 1% with acetic acid pH 3.5.

Chitosan was extracted from squid pens (*Doryteuthis* spp.), as described by Horn et al. (2015) [48]. The squid pens were previously washed, dried, crushed and sifted to obtain particles smaller than 0.25 mm. Demineralization and deproteinization steps were performed using dilute solutions of HCl (at room temperature for 2 h) and NaOH (at 80 °C for 1 h), respectively. Beta-chitin was deacetylated with a concentrated sodium hydroxide solution (40% NaOH, *w*/*w*) at 80 °C for 3 h in nitrogen atmosphere. The chitosan powder was obtained after washing and drying. The chitosan powder was solubilized in acetic acid, pH 3.5, to obtain a 1% chitosan gel (*w*/*w*). The degree of acetylation (11.2%) and molecular weight (1.6 × 10^5^ g mol^−1^) were determined by nuclear magnetic resonance (NMR) spectroscopy and capillary viscosimetry, respectively.

### 2.2. Scaffold Preparation

The scaffold was prepared by mixing anionic collagen with chitosan (3:1 ratio) under mechanical stirring at 800 rpm (Fisaton^®^, mod. 715, São Paulo, Brazil). The gel was placed in Teflon^®^ molds (São Paulo, Brazil), frozen in liquid nitrogen and lyophilized. These scaffolds were neutralized using ammonia vapor for 8 h.

### 2.3. Characterization

Differential scanning calorimetry (DSC): Thermal stability of the scaffold was determined by differential scanning calorimetry (DSC-2010^®^ equipment, TA Instruments, New Castle, DE, USA). Twenty mg of the sample were placed in a hermetically sealed aluminum support under an N_2_ flow (80 mL min^−1^). The temperature range was 25–120 °C, with a heating rate of 10 °C min^−1^. The temperature of denaturation was given from the inflection point.

Attenuated total reflectance Fourier transform infrared spectroscopy (ATR-FTIR): Samples of collagen, chitosan and collagen/chitosan (3:1) scaffolds were analyzed in an ATR_FTIR Bruker Alpha Platinum-ATR a range of 4000–400 cm^−1^, with 64 scans and a resolution of 2 cm^−1^.

Scanning electron microscopy (SEM) analysis: The surface and cross-section were analyzed by SEM using a Zeiss Leo 440 scanning electron microscope (Cambridge, UK) with Oxford detector (model 7060, Cambridge, UK) operating with a 20 kV electron beam. The scaffold was coated with gold in a BAL-TEC MED 020 Coating System (Bal-Tec^®^, Balzers, Liechtenstein), with a chamber pressure of 2.00 × 10^−2^ mbar, 60 mA current and 0.60 nm s^−1^ deposition rate. The pore size of the scaffold was determined using Martin’s diameter (The pore size of the scaffold was determined using Martin’s diameter method [48] and at least 30 pores were measured with ImageJ^®^ software (1.48 version, Bethesda, MD, USA).

Porosity was determined following the procedure adapted from Nwe et al. (2009) [49]. The scaffolds were cylindrical in shape, with average diameter of 5 mm and average thickness of 0.44 cm. A 5 mL sample of ethanol was weighed (w_1_) and the scaffolds were immersed in it; after 24 h, the scaffolds were removed and the ethanol was weighed again (w_2_). Porosity was determined in triplicate, according to the equation: P(%) = (V_2_/V_1_) × 100, where V_2_ = (w_1_ − w_2_)/ρ and ρ is the ethanol density.

### 2.4. Sterilization of Scaffolds/Matrices

The samples of the anionic collagen/chitosan scaffolds were sterilized with ethylene oxide at Sterilization Center (ACECIL^®^ Com. Ind. Ltd., Campinas, Brazil).

### 2.5. Obtention of Mesenchymal Stem Cells (MSCs) and Cell Expansion

Commercial MSCs (Lonza Bioscience^©^, Walkersville, MD, USA) derived from human dental pulp were provided and manipulated by R-Crio Células Tronco (Campinas, Brazil), where the technicians of that laboratory expanded these cells and, later, associated them with the scaffold. This cell line were chosen solely based on being human cells from an established primary culture.

The cells were thawed at room temperature and transferred to a 25-cm² culture flask (NEST, Jiangsu, China). The culture medium consisted of Dulbecco’s Modified Eagle’s Medium (DMEM)—low glucose (Sigma^®^, Riverside, CA, USA), 100 µM ascorbic acid (Sigma, CA, USA), penicillin–streptomycin solution (Thermo-Fisher^®^, Massachusetts, MA, USA) and 10% human serum (Sigma^®^, Riverside, CA, USA). The flasks with cells were incubated at 37 °C in a 5% CO_2_ atmosphere and the medium was changed every 48 h.

### 2.6. Biomaterial Combination

After the monolayer reached 70% of confluence, the cells were detached by enzymatic action (TrypLE Express Enzyme, Gibco^®^, Thermo-Fisher, Waltham, MA, USA). After complete detachment, the cells were washed and counted by the Trypan Blue exclusion method. Next, 5 × 10^4^ cells suspended in 10 µL of the same expansion medium were seeded on the biomaterial in a 12-well plate (Figure S1, see Supplementary Materials). The plate was further incubated at 37 °C in a 5% CO_2_ atmosphere for 48 h.

### 2.7. Experimental Design

**Figure 1:**
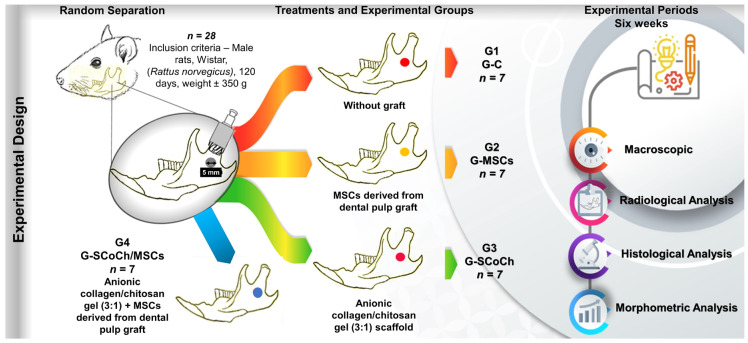
Experimental design. Twenty-eight male rats (*Rattus norvegicus*) aged 120 days were included. In all animals, a circular bone defect 5 mm in diameter was surgically created in the right mandibular ramus. The animals were separated into four groups of seven animals each: G1 (G-C), without graft (clot only); G2 (G-MSCs), defect filled with MSCs derived from dental pulp; G3 (G-SCoCh), defect filled with anionic collagen/chitosan gel scaffold; G4 (G-SCoCh/MSCs), defect filled with anionic collagen/chitosan gel scaffold + MSCs. All animals were euthanized 6 weeks after surgery and macroscopic, radiological, histological and morphometric evaluations were performed.

Twenty-eight male Wistar rats (*Rattus norvegicus*), 4 months old and weighing on average 350 g, were used. The animals were maintained under adequate conditions in the Animal House of the Jundiaí Medical School (FMJ), Jundiaí, Brazil. The Ethics Committee on Animal Experimentation of FMJ approved the project (Protocol 22511540, approval date 14 February 2022). The rats were submitted to a surgical procedure for creation of an experimental defect in the right mandibular ramus and were divided into four groups of seven animals each (Figure 1):

- G1 (G-C): mandibular defect filled with the blood clot of the defect but did not receive the graft (control group);
- G2 (G-MSCs): mandibular defect seeded with 5 × 10^4^ DPSCs suspended in 10 µL culture medium;
- G3 (G-SCoCh): mandibular defect grafted with the anionic collagen/chitosan gel (3:1) scaffold;
- G4 (G-SCoCh/MSCs): mandibular defect grafted with the anionic collagen/chitosan gel (3:1) scaffold and seeded with 5 × 10^4^ DPSCs.

### 2.8. Surgical Technique for Creation of the Mandibular Defect

The animals were anesthetized by intramuscular administration of xylazine (Vetaset^®^, Fort Dodge Saúde Animal Ltd., Campinas, Brazil) and ketamine (Dopalen^®^, Agibrands of Brazil Ltd., Campinas, Brazil) (1:1) at a dose of 0.1 mL/100 g body weight into the gluteus muscle [9]. In addition, tramadol hydrochloride (0.01 mg/100 g body weight) was injected subcutaneously into the animal’s back. After the confirmation of anesthesia, the animals were placed in left lateral decubitus and the right hemiface was shaved, followed by local asepsis with 2% chlorhexidine digluconate solution. Next, an oblique skin incision was made in the middle third of the mandibular area to identify the local muscles, which were sectioned and separated to expose the right ramus of the mandible.

A circular bone defect measuring 5 mm in diameter was created using a trephine drill coupled to a mini-motor (BELTEC^®^ LB-100, Araraquara, Brazil) [50]. The defect was filled as described for the experimental groups, except for G1 (control) in which the bone defect was filled only with the clot triggered by the injury; therefore, the scaffolds filled the entire bone defect created. At the end of the surgical procedure, the soft tissues were repositioned and sutured. Each animal remained separate in the cage and received a special powdered diet (AIN-93M diet, soy protein isolate), which was mixed with filtered water to obtain a pasty consistency in order to facilitate feeding during the postoperative period. Water was available ad libitum.

During the postoperative period, the rats received daily intramuscular injections of 0.1 mg/100 g body weight of Pentabiotic Veterinário Pequeno Porte antibiotic (Fort Dodge^®^, Campinas, Brazil) for one week [16]. In addition, meloxicam as anti-inflammatory and tramadol hydrochloride (0.01 mg/100 g body weight) as analgesic were administered subcutaneously on the back for one week. Rifamycin spray (Rifocina^®^, Sanofi-Aventis Pharmaceutical Ltd., São Paulo, Brazil) was applied externally to the defect area. Paracetamol diluted in the animal’s water was also provided during the postoperative period and the animals were constantly monitored to assess their behavior. All animals responded adequately to the pre- and postoperative procedures and there was no need for replacement of animals in the groups.

### 2.9. Macroscopic, Radiological and Histomorphometric Analysis of the Defect Area

Six weeks after surgery, euthanasia was performed in a silent environment and away from other animals, using barbiturate thiopental at the dosage for rats (150 mg/kg) as follows: Sodium Thiopental 2.5% (Thiopentax^®^, Cristália, Itapira, Brazil) intraperitoneally, applied in the abdominal quadrant inferior left of the animal, associated with the local anesthetic lidocaine hydrochloride (Xylestesin^®^, Cristália, Itapira, Brazil), at a dosage of 10 mg/kg. The mandible was immediately removed and photodocumented to analyze the clinical conditions of the defect area. The samples were fixed in formaldehyde solution and submitted to image analysis by digital radiography (AJEX-240 Diagnostic X-Ray System: 40–120 kVp, 0.4–100 mA, D-125 Inserted Xray Tube, and Pixx1717 flat panel detector, Pixxogen).

After digital radiography, the collected bone pieces were washed in water for 24 h and submitted to the demineralization process using a solution of ethylenediaminetetraacetic acid (EDTA), a solution containing 4.13% of tritiplex III (Merck KGaA^®^, Hessen, Germany) and 0.44% sodium hydroxide (Labsynth^®^, São Paulo, Brazil). The decalcifying solution was changed weekly and, to assess the complete decalcification, radiographs were taken with periapical film Insight Adult IP-21 F-Speed Carestream (Carestream Health^®^, Rochester, NY, USA) for an approximate period of 6 weeks. After completing this phase, the pieces were dehydrated in an increasing series of ethyl alcohol, cleared in xylene and embedded in Histosec paraffin (Merck^®^, Hessen, Germany).

Semi-serial sections (5 μm) were cut along the bone defect. The histological sections were stained with Masson’s trichrome for the characterization of new bone formation and with Picrosirius red (saturated aqueous solution of picric acid plus 0.1 g Sirius red F3B-Bayer^®^) for analysis of the fibrillar components of ECM by polarized light microscopy. The histological sections used for staining and subsequent qualitative and quantitative analysis were made in the center of the 5 mm diameter defect and also at the medium distance between the center and the peripheral region (Figure S2).

To define the newly formed bone, the Motic Images Plus 2.0 ML software was used to quantify the percentage of newly formed bone in the defect in the right mandibular ramus. These data were entered into the BioEstat 5.3 software and ANOVA followed by the Tukey test was applied for statistical comparison between the groups studied, adopting a level of significance of *p* < 0.05.

## 3. Results and Discussion

### 3.1. Analysis of Scaffolds

The scaffold was white and had a homogeneous surface. The scaffold was characterized by DSC, ATR-FTIR, SEM and porosity assay. The denaturation temperature (Td) for the collagen present in the scaffold was 48.7 °C. The collagen extraction procedure used promotes selective hydrolysis of the collagen carboxyamide groups, increasing the negative charges of the biopolymer [51]. This increase in negative charges improves the biocompatibility of collagen [51,52,53]. Thermal analysis was performed to evaluate the integrity of the collagen triple helix and to confirm the presence of anionic type I collagen. The scaffold developed with collagen and chitosan (ratio of 3:1) had a Td similar (48.7 °C) to that of the collagen scaffold without the addition of chitosan (47.9 °C) obtained by Massimino et al. (2020) [54]. This result demonstrates the integrity of the triple helix and the presence of type I collagen.

**Figure 2:**
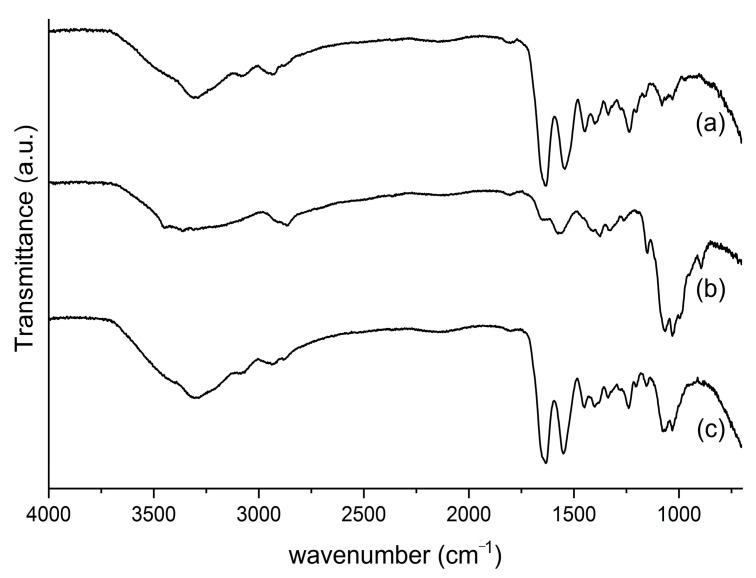
ATR-FTIR spectra of (a) collagen, (b) chitosan and (c) collagen/chitosan scaffolds.

The ATR-FTIR spectra of the bovine collagen, chitosan and collagen/chitosan scaffold are shown in Figure 2. The ratio between the absorbance at 1235 and 1450 cm^−1^ shows the integrity of the collagen triple helix, with values close to 1.0 indicating that the collagen did not undergo denaturation; and in this case, the bovine collagen had a ratio of 0.96. The typical amide bands can be observed, like amide A band in the 3720–3120 cm^−1^ region, amide B in the 3115–2815 cm^−1^ region, amide I at 1633 cm^−1^, amide II at 1549 cm^−1^ and amide III at 1243 cm^−1^. These bands agree with the collagen extraction processes shown in Figure 2a [55]. Characteristic chitosan bands were observed at 1073 cm^−1^ and 1033 cm^−1^, referring to the −CO group in CH_2_−OH and overlap with the −CO group in the ether−COC−bond (pyranose ring) [56] (Figure 2b).

The bands shown in the ATR-FTIR spectrum of the collagen/chitosan scaffold are consistent with its precursors (Figure 2a,b) and the literature [55,56].

**Figure 3:**
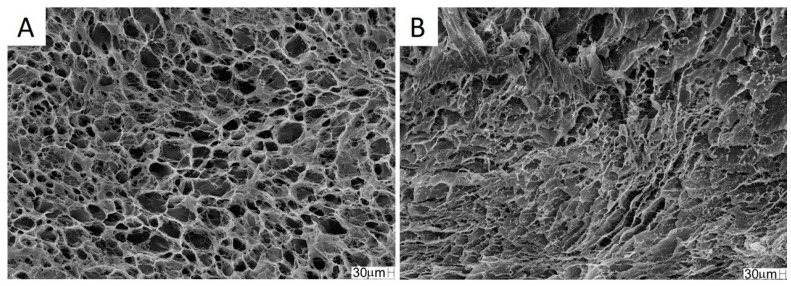
SEM photomicrographs of the collagen/chitosan scaffold. (**A**) superficial section; (**B**) cross-section. Magnification: 200×.

The SEM photomicrographs showed superficial pores in the scaffold, and interconnected pore spaces can be seen in the inner structure of the scaffold (Figure 3A,B). The pore size, as by SEM analysis with ImageJ^®^, was 55 ± 16 μm and porosity was 95.0 ± 1.5%.

There is no consensus in the literature regarding the ideal pore size for bone regeneration. According to Silva et al. (2022) [18], pores between 20.6 ± 3.2 and 13.5 ± 3.3 μm contribute to the regeneration of defects in long bones. Battafarano et al. (2021) [57] suggested pores between 200 and 350 μm for bone growth. According to Zhao et al. (2019), scaffolds with pores between 300 and 900 μm are suitable for bone reconstruction. Zhang et al. (2018) [58] concluded that a smaller diameter pore aids in cell adhesion. These smaller pores also promote a capillary force, which assists in the retention of physiological fluids and cell permeation into the scaffold, allowing greater integration of the biomaterial with the tissue [59].

Pores smaller than 100 μm contribute to cell adhesion and bone regeneration [60]. The scaffold selected for this study had pores of 55 ± 16 μm, theoretically suitable for the proposed use, as this porosity would allow for better cell adhesion when used in conjunction with stem cells. However, no significant improvement in bone regeneration was observed, and this fact may have occurred because the stem cells were seeded on the scaffold at the time of surgery and did not have time to adhere sufficiently to the material.

In addition to pore size and interconnectivity, porosity is also an important factor for bone regeneration. The percentage of porosity influences the absorption of exudates, the colonization rate, the cell structure and the process of angiogenesis [61]. High porosity is required for bone growth, ideally ranging between 80 and 90% [62]. The porosity percentage of the scaffold obtained was within this range (95.0 ± 1.5%), indicating that it has the appropriate characteristics for use in bone regeneration.

### 3.2. Macroscopic and Radiological Analysis of Bone Defects

This study evaluated the capacity of polymeric collagen/chitosan scaffolds seeded with DPSCs to provide an osteogenic stimulus for bone neoformation during treatment of mandibular bone defects experimentally induced in rats. The results obtained were compared with data reported by other researchers and some important differences and similarities were observed. Within this context, we hope that the results obtained can contribute to tissue engineering in regenerative medicine and dentistry and help surgeons choose the best scaffold for the treatment of craniomaxillofacial injuries. For this purpose, the availability and favorable use properties of natural polymers in tissue engineering must be considered [63], as well as DPSCs whose extraction process is simple and which can be easily obtained from deciduous or permanent teeth [64].

**Figure 4:**
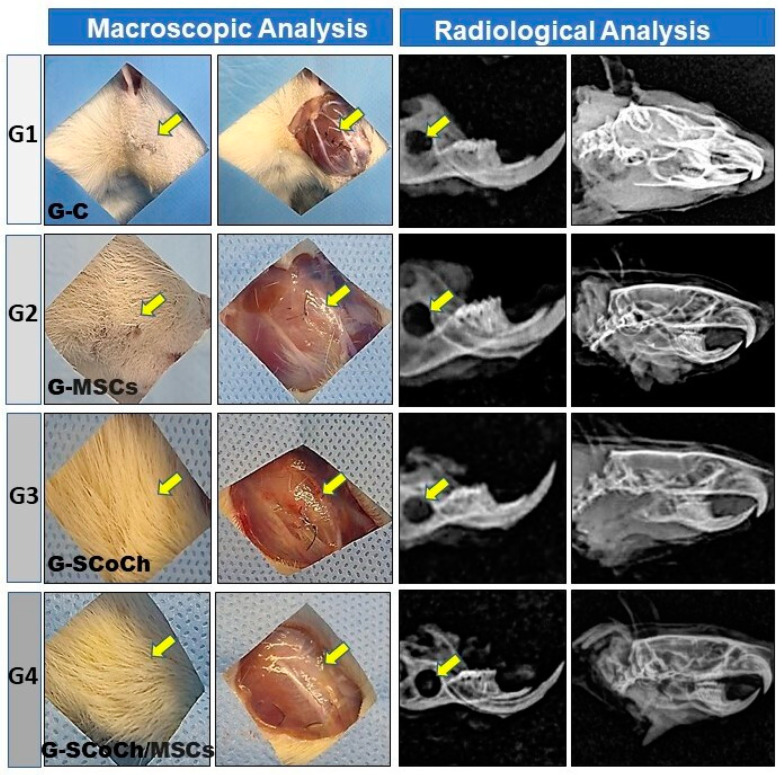
Macroscopic and radiological images of the defect area in rats of the groups studied (G1 to G4). Note the growth of hair and the absence of signs of infection (yellow arrows). The radiological images show the persistence and radiolucency of the defect in the mandibular ramus and the absence of signs of bone rarefaction in adjacent areas.

The results regarding the clinical conditions of the animals 6 weeks after surgery showed good recovery of all rats as demonstrated by adequate skin healing and normal hair growth in the defect area, absence of clinical signs of inflammatory processes, and normal behavior and feeding of the animals. In the implanted groups (G2, G3, G4), there were no signs of subcutaneous infection or infection in deeper anatomical planes that would indicate immune rejection of DPSCs or of the scaffolds used as implants. The soft tissues in the defect area had healed well, with no formation of adhesions (Figure 4).

The positive clinical results of the animals can be attributed to the fact that all animal use experimental protocols were followed and to the pre- and postoperative care and sterilization of the scaffolds and surgical field, as carried out in other studies on the regeneration of craniofacial injuries [8,9,11,17,65,66]. These macroscopic observations of satisfactory recovery of the animals were confirmed by radiological analysis, which demonstrated the absence of bone rarefaction, secondary fractures, pseudarthrosis, osteomyelitis, abnormalities and carcinogenic processes in the defect area. The mandible exhibited normal morphology and good radiopacity and the experimentally induced defect persisted, showing radiopaque borders and a radiolucent center (Figure 4).

Successfully used in different regenerative medicine applications, ECM scaffolds can be obtained by decellularization of manipulated tissue [67]. Thus, collagen, which is the main component of ECM, was used in the present study. This collagen was extracted from bovine tendons by selective hydrolysis of the carboxyamide groups, a process that increases the negative charges on the biopolymer [51]. This increase in negative charges improves the biocompatibility of collagen [51,52] and also promotes decellularization of the tissue used for collagen extraction, thereby contributing to minimizing possible immune rejection of the scaffolds when implanted into the host tissue.

According to Horn et al. (2009) [59], the duration of alkaline hydrolysis during the preparation of anionic collagen influences the properties and porosity of the scaffold. Other decellularization methods have been described in the literature. For example, Wollmann et al. (2019) [68] used sodium dodecyl sulfate and ethylenediaminetetraacetic acid to remove cells from the human pericardium, maintaining the properties and mechanical integrity of ECM. Thus, decellularization of the native tissue is important for the process of biocompatibility when used as an implant of natural origin in order to avoid inflammatory reactions in the host tissue.

Biomaterials used in tissue engineering must meet certain requisites when implanted into the host tissue, which include being non-toxic, non-immunogenic, biodegradable and able to simulate the microenvironment necessary for cell growth, proliferation and migration in order to promote the formation of new tissues [69]. Confirming the biocompatibility of the scaffolds used in this study, in addition to the absence of macroscopic and radiological signs of infection, the histological results showed the absence of an inflammatory infiltrate and fibrotic tissue in the mandibular defect area of rats in G3 and G4 that received the anionic collagen/chitosan scaffolds.

Based on the above considerations and the macroscopic, radiological and histological results that indicate biocompatibility of the scaffolds and DPSCs used in this study, histomorphometric analysis of new bone formation in the defect area was performed to confirm the osteogenic efficacy of the standardized protocol.

### 3.3. Microscopic Results and Morphometric Analysis of Newly Formed Bone Percentage in the Defect Area

Polymeric membranes with or without cellular or biological mediators have received special attention in biomedical engineering and dental, oral and craniofacial regenerative medicine for their role as scaffolds in cell therapies and as slow-release devices of drugs, biomolecules and growth factors that are important for the in vitro development of tissue and organs [12,15,70]. Collagen has the advantage that it can be modified chemically by alkaline hydrolysis, resulting in anionic collagen that is negatively charged at pH 7.4 [51]. Chitosan is an abundant polysaccharide in nature that is obtained from deacetylated chitin isolated from the exoskeleton of crustaceans, fungi and insects [71].

The combined use of anionic collagen biopolymer and chitosan has been shown to stimulate vascular formation and to improve the three-dimensional structure of the scaffold, enabling cell migration [72]. In addition, its combination with DPSCs stimulates osteoinduction, thus representing a promising protocol for tissue bioengineering [69]. Horn et al. (2009) [59] prepared chitosan/collagen blends at a ratio of 1:1 (*w*/*w*). Chitosan was obtained by deacetylation of squid chitin and porcine collagen was obtained from the serosa using alkaline hydrolysis of different durations. The authors demonstrated that this protocol interferes with pore size. In the present study, we prepared collagen from bovine tendon and subjected it to alkaline hydrolysis [48] for 72 h at room temperature. We thus obtained anionic collagen samples with porosity of 95.0 ± 1.5%, a percentage that is sufficient to indicate the material for bone regeneration.

Considering the advantages of alkaline hydrolysis for natural polymers described by Bet et al. (2001) [51], studies have demonstrated the favorable use properties of anionic collagen applied to bone defects experimentally induced in rats. The results showed biocompatibility with the host tissue since native cells of the tissue were removed during the fabrication of these materials, with anionic collagen mimicking the ECM. In addition, collagen stimulated cellular and vascular regeneration, promoting the formation of new tissue that resembled the original bone [8,17,73].

**Figure 5:**
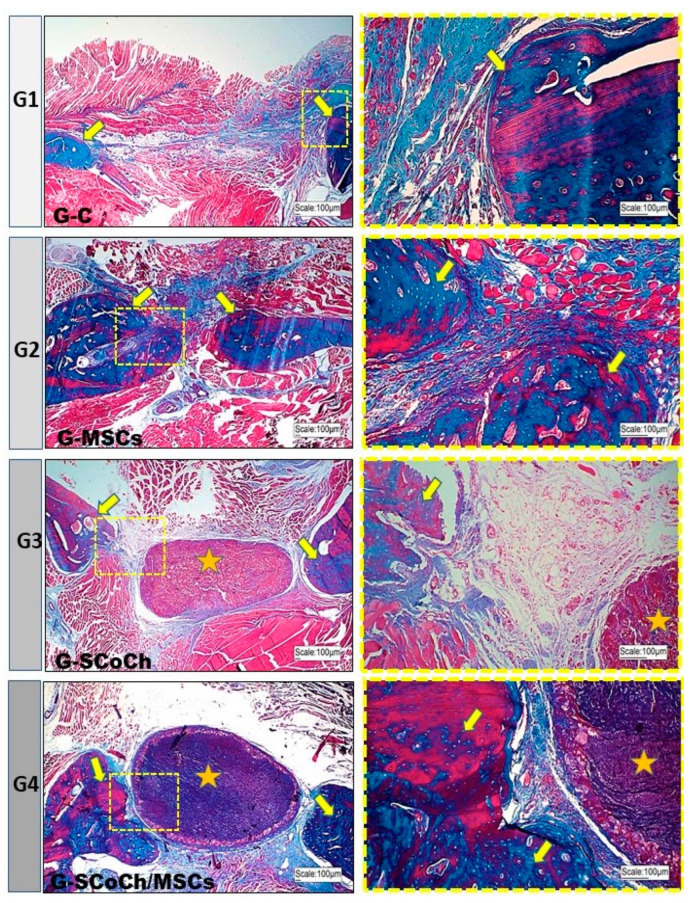
Light microscopy images of the mandibular defect area in the groups of rats studied (G1 to G4). Note the new bone formation (yellow arrows) starting from the borders of the defect. In the center of the defect, there was a predominance of abundant connective tissue in G1, bone formation in G2 and persistence of the scaffold (yellow asterisk) in G3 and G4. In G4, there was closer interaction between bone and scaffold. Masson’s Trichrome staining. In blue, new bone and connective tissue.

In this experimental protocol using rat mandible, bone formation occurred from the borders of the defect towards the polymer in the group grafted with anionic collagen/chitosan scaffolds (G3); however, there was no direct bone-implant interaction due to the presence of interposed connective tissue. In G4 that received the same scaffold but seeded with DPSCs in vitro, greater bone formation closer to the biomaterial was observed, with little connective tissue at the bone-implant interface (Figure 5). However, complete repair of the defect did not occur within the 6 weeks of the experiment, as also observed in G3. More promising results in terms of bone formation were expected for G4; however, the fact that in vitro cell expansion takes a long time must be considered [64], as well as the embryological origin of the mandible, which derives from intramembranous ossification, with bone repair thus being slower when compared to endochondral bones. Intramembranous ossification, vascularization and surrounding soft tissues must be considered [3,74] since these factors are determinants for the choice of membrane to be used as implant.

In G2 animals seeded only with DPSCs, bone formation occurred towards the center of the defect, probably because these cells were introduced into the entire space of the defect. In the control group (G1), there was little bone formation at the borders of the defect, with the largest part of the defect being filled predominantly with connective tissue (Figure 5). These results are similar to that obtained by Jégoux et al. (2010) [75], who studied the healing of mandibular defects in dogs. After 24 weeks, the authors noted the absence of spontaneous healing in the control group, with bone formation occurring only at the borders of the defect. Studying the osteogenic potential of a cross-linked type I bovine collagen membrane in the healing of mandibular bone defects applying the concept of guided bone regeneration, Zahedi et al. (1998) [50] also observed limited amounts of newly formed bone at the borders of the defects in the control group. In 90- and 180-day-old animals, the grafted defects were completely closed. Similar results have been reported by McAllister and Haghighat (2007) [14] who stated that, in cases of large defects, bone formation occurs only in the marginal stable zone, while the central zone is filled with disorganized loose connective tissue. Thus, a stimulus provided by a natural or synthetic graft is required to promote the osteoconduction, osteogenesis and osteoinduction necessary for bone repair.

Soares et al. (2016) [76] tested a biomembrane produced from a solution of collagen and chitosan (2:1) immersed in calcium aluminate and seeded with DPSCs. The results showed an increase in alkaline phosphatase activity and the biomembrane induced the differentiation of pulp cells into odontoblast-like cells. The same group reported similar results in a subsequent study [77]. Chamieh et al. (2016) [78] found that the combination of DPSCs and a collagen scaffold promoted the repair of cranial defects.

**Figure 6:**
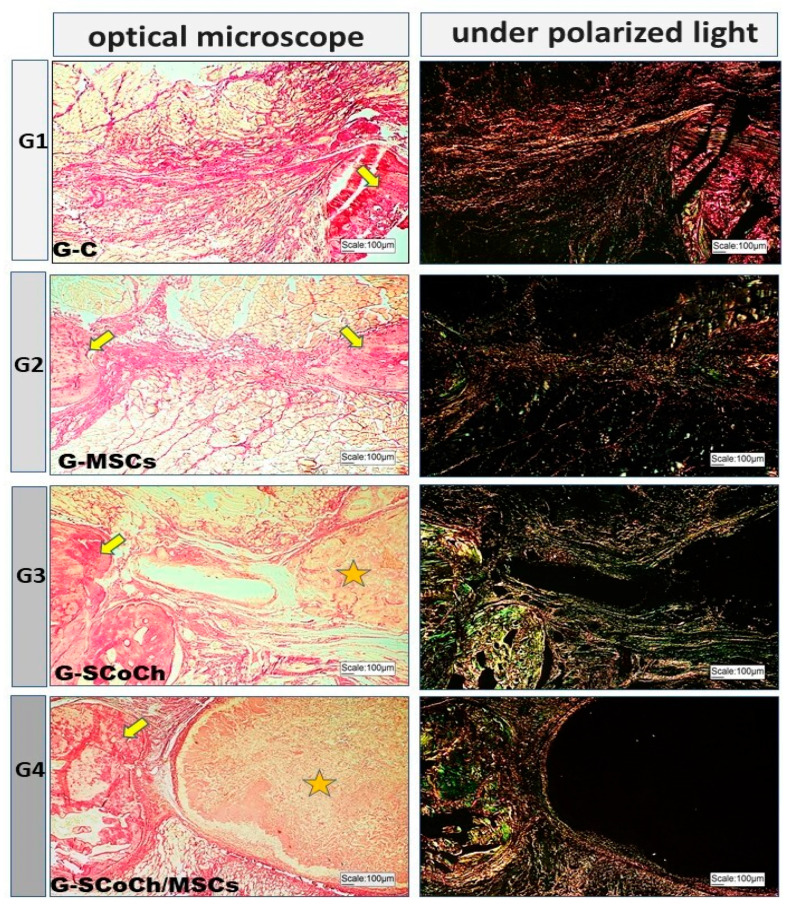
Light microscopy and respective polarized light images of the groups studied using Picrosirius red staining. Birefringence of the extracellular matrix is observed in the bone defect area, in bone formed at the borders of the defect (yellow arrows) and around the scaffolds (asterisk) in G3 and G4.

In all groups studied, the newly formed bone showed histological characteristics of immature tissue and the presence of osteoid matrix (Figure 5), with an imaging characteristic of low radiopacity (Figure 4), generally due to the degree of mineralization during the experimental period that we stipulated from 6 weeks. Also in Figure 4, radiolucent areas can be seen in regions of the defect, which were not completely filled by new bone tissue. Van Leeuwen et al. (2012) [79] obtained good results, in a study similar to ours, but with experimental periods of up to 12 weeks, using membranes in the guided bone regeneration (GBR) technique; and the poly (trimethylene carbonate, PTMC) membrane showed itself suitable for GBR. In Figure 6, with Picrosirius red staining, a birefringence transition of the collagen fibers from orange-red (more visible in G1) to greenish-yellow (G2–G4) can be observed, in which the thinner and disorganized fibers progress to more organized and thicker [11,73].

To confirm the stimulus potential of the implants used in this study, the percentage of newly formed bone in the defect area was analyzed morphometrically. Histological sections were made in the center of the 5 mm diameter defect and also in the middle distance between the center and the peripheral region (Figure S2). The percentage of new bone formed in groups G1, G2, G3 and G4 is shown in Table 1.

Statistically, all possible comparisons showed significant differences between groups (*p* < 0.05). These results suggest that a stimulus is necessary for bone repair in critical mandibular defects since the lowest value was observed in the control group (G1). Among the groups studied, G2 exhibited the greatest bone formation at the periphery and in the center of the defect, suggesting that MSCs exert an osteogenic effect which, however, is not sufficient to promote complete regeneration within the period of 6 weeks after surgery standardized in this study. Jégoux et al. (2010) [75] used bone marrow grafts in the center of the implants placed in mandibular defects of segmental mandibulectomy in dogs and observed new bone formation after 16 weeks, supporting the theory that bone marrow cells induce MSCs to migrate within the bone defect and differentiate into an osteoblastic lineage. The same was observed by de Oliveira e Silva et al. (2012) [19], who evaluated the healing of critical-sized bone defects on rabbits’ skull by using a mix of mineralized xenograft and autologous bone marrow. Overall, research with rats shows better bone formation in groups with membrane-treated mandibular defects compared to untreated animals [50,80].

In the present study, despite the good results observed in the grafted groups (G2, G3, G4) compared to the control group (G1), complete repair did not occur probably because a postoperative period of 6 weeks was standardized in order to determine the speed of bone formation in response to the protocol used [81,82]. Thus, it is important to observe the long-term results in research with grafting of barrier membranes in animals, because it is still unknown whether long-term mineralization occurs in the soft tissue layer formed under the membrane [3]. In addition, there were ethical issues since the study was conducted in accordance with the principles of the NC3Rs [83], thus using the smallest number of animals as possible; this factor also led us not to increase the number of groups studied. Further studies with different postoperative periods are therefore necessary to evaluate bone repair in rat mandibles using polymers and cell therapy; the intramembranous embryological origin of bones that interferes with bone regeneration must also be considered [84].

Mechanical properties, degradation capacity and the porosity necessary for vascular and bone growth must also be analyzed when selecting a biomaterial [3,85,86]. In the present study, the anionic collagen/chitosan scaffold persisted in the bone defect area 6 weeks after surgery, indicating long-term biodegradability of the material. The calcification process takes longer in the case of hard tissues such as bone; thus, the scaffold must provide long-term mechanical support that enables the host tissue to accept the implant, promoting interaction and cell growth [87].

According to the concept of guided bone regeneration in maxillofacial surgeries, biomaterials are applied to bone defects in order to maintain a space between the bone defect and the periosteum and to assist with bone formation. Ideally, osteoprogenitor cells should colonize the space beneath the bone defect; however, these cells have a slower growth rate than epithelial and connective tissue cells. To prevent the bone defect from being occupied by epithelial cells and connective tissue, a membrane (resorbable or non-resorbable) is used to cover the graft [88,89].

Polymeric materials are considered osteoconductive and maintain space for cell migration to the bone defect in order to induce the regeneration process [15]. The chitosan/collagen mixture provides an ECM-like substrate and serves as a support for the proliferation of human dental pulp cells and expression of odontoblastic phenotypes, thus promoting good deposition of mineralized matrix [76]. Thus, in the present study, the anionic collagen/chitosan scaffold was inserted into the mandibular defects of rats and was not used as a coating membrane according to the concept of guided bone regeneration. In this regard, it was observed that G4 resulted in higher bone formation levels when compared with G3, suggesting that the collagen/chitosan matrix is suitable for seeding with DPSCs. The bone percentage was greater in the groups implanted with the scaffolds (G3, G4) compared to the control group (G1), which might suggest that the collagen/chitosan matrix avoided the proliferation of soft tissue cells from the surrounding tissues to the center of the defect.

In Figure 5 and Figure 6, the presence of the scaffold (highlighted in yellow stars) can be seen in the implanted site, in G3 and G4. However, as G2 (51.17 ± 2.98) performed better than G4 (45.26 ± 1.04), the scaffold used may not have contributed to the formation of new bone tissue. There is a possibility that, in addition to porosity, the degree of biocompatibility of the scaffold for DPSCs may be an explanation for the reduced repair detected in G4 vs G2. Pore size and geometry play a significant role in tissue regeneration and strongly influence cell adhesion, cell-cell interaction and cell transmigration across the membrane [90]. Thus, perhaps the collagen/chitosan matrix used in this study did not contribute to cell migration and adhesion. Therefore, its use can be tested in the future as a membrane in guided bone regeneration (GBR).

In addition, the combination of guided bone regeneration technique with DPSCs would help develop a treatment protocol capable of enhancing cell function and mimicking the physiological reactions of bone tissue in order to accelerate the process of bone regeneration in maxillofacial traumas. All of these factors will contribute to more personalized treatment and a faster and more satisfactory recovery of the patient.

A possible limitation of the study can be considered, immunohistochemical reactions with bone markers for evaluation of protein expression could correlate with the histological findings of the repair process. Also, the lack of analyses prior to this study on the biocompatibility of the scaffold for seeded DPSCs can be considered a limitation.

## 4. Conclusions

This pre-clinical experimental protocol was carried out in order to evaluate the in vivo effects of the combination of anionic collagen/chitosan scaffold with or without DPSCs in the repair of mandibular bone defects in rats. The scaffold used showed osteoconductivity, no foreign body reaction, malleability and ease of manipulation, but did not obtain promising results for association with DPSCs. The scaffold-filled groups (G3 and G4) had a higher percentage of new bone formed in the defect area than the control (G1, clot only), but the presence of this type of scaffold is less effective than DPSC alone in formation of new bone (G2).

## Figures and Tables

**Table 1:** Percentage of new bone formed in each group within 6 weeks post-surgery.

G1 (G-C)	G2 (G-MSCs)	G3 (G-SCoCh)	G4 (G-SCoCh/MSCs)
16.20 ± 2.83 a	51.17 ± 2.98 b	31.31 ± 3.40 c	45.26 ± 1.04 d

Different lowercase letters, line comparison, G1 (G-C) vs. G2 (G-MSCs) vs. G3 (G-SCoCh) vs. G4 (G-SCoCh/MSCs) in period of 6 weeks (a ≠ b ≠ c ≠ d) indicate a statistically significant difference. Values defined as the mean ± standard deviation. Tukey test at *p* < 0.05.

## Data Availability

The data presented in this study are available on request from the corresponding author.

## Supplementary Materials

The following supporting information can be downloaded at: https://www.mdpi.com/article/10.3390/jfb14070357/s1, Figure S1: (A) Scaffold with caliper; (B–C) Stem cell seeding on scaffold; (D) Scaffold in position on the defect created in the mandible, Figure S2: Three levels of histological sections (yellow lines) used for staining and subsequent qualitative and quantitative analysis: center of the defect and medium distance between the center and the peripheral region.

## References

[1] Scannavino F.L.F., dos Santos F.S., Neto J.P.N., Novo L.P. (2013). Epidemiological analysis of maxillofacial trauma of an emergency service. Rev. Cir. e Traumatol. Buco-Maxilo-Facial.

[2] Parashar A., Sharma R.K. (2013). Unfavourable outcomes in maxillofacial injuries: How to avoid and manage. Indian J. Plast. Surg..

[3] Dimitriou R., Mataliotakis G.I., Calori G.M., Giannoudis P.V. (2012). The role of barrier membranes for guided bone regeneration and restoration of large bone defects: Current experimental and clinical evidence. BMC Med..

[4] Gaihre B., Uswatta S., Jayasuriya A. (2017). Reconstruction of Craniomaxillofacial Bone Defects Using Tissue-Engineering Strategies with Injectable and Non-Injectable Scaffolds. J. Funct. Biomater..

[5] Janicki P., Schmidmaier G. (2011). What should be the characteristics of the ideal bone graft substitute? Combining scaffolds with growth factors and/or stem cells. Injury.

[6] de Rosso M.P.O., Buchaim D.V., Pomini K.T., Della Coletta B.B., Reis C.H.B., Pilon J.P.G., Júnior G.D., Buchaim R.L. (2019). Photobiomodulation therapy (PBMT) applied in bone reconstructive surgery using bovine bone grafts: A systematic review. Materials.

[7] Pomini K.T., Buchaim D.V., Andreo J.C., de Rosso M.P.O., Della Coletta B.B., German Í.J.S., Biguetti A.C.C., Shinohara A.L., Rosa Júnior G.M., Shindo J.V.T.C. (2019). Fibrin sealant derived from human plasma as a scaffold for bone grafts associated with photobiomodulation therapy. Int. J. Mol. Sci..

[8] Munhoz M.A.S., Hirata H.H., Plepis A.M.G., Martins V.C.A., Cunha M.R. (2018). Use of collagen/chitosan sponges mineralized with hydroxyapatite for the repair of cranial defects in rats. Injury.

[9] Cunha F.B., Pomini K.T., de Plepis A.M.G., da Martins V.C.A., Machado E.G., de Moraes R., de Munhoz M.A.E.S., Machado M.V.R., Duarte M.A.H., Alcalde M.P. (2021). In Vivo Biological Behavior of Polymer Scaffolds of Natural Origin in the Bone Repair Process. Molecules.

[10] De Marchi T., Francio F., Ferlito J.V., Weigert R.M., de Oliveira C.A., Merlo A.P., Pandini D.L., Junior B.A.P., Giovanella D.F., Tomazoni S.S. (2020). Effects of photobiomodulation therapy combined with static magnetic field (PBMT-sMF) in patients with severe COVID-19 requiring intubation a pragmatic randomized placebo-controlled trial. medRxiv.

[11] Pandini F.E., Miyauchi Kubo F.M., De Guzzi Plepis A.M., Da Conceição Amaro Martins V., Da Cunha M.R., Silva V.R., Hirota V.B., Lopes E., Menezes M.A., Pelegrine A.A. (2022). In Vivo Study of Nasal Bone Reconstruction with Collagen, Elastin and ChitosanMembranes in Abstainer and Alcoholic Rats. Polymers.

[12] Koons G.L., Diba M., Mikos A.G. (2020). Materials design for bone-tissue engineering. Nat. Rev. Mater..

[13] Lin W., Mashiah R., Seror J., Kadar A., Dolkart O., Pritsch T., Goldberg R., Klein J. (2019). Lipid-hyaluronan synergy strongly reduces intrasynovial tissue boundary friction. Acta Biomater..

[14] McAllister B.S., Haghighat K. (2007). Bone Augmentation Techniques. J. Periodontol..

[15] Wu D.T., Munguia-Lopez J.G., Cho Y.W., Ma X., Song V., Zhu Z., Tran S.D. (2021). Polymeric scaffolds for dental, oral, and craniofacial regenerative medicine. Molecules.

[16] Chacon E.L., Bertolo M.R.V., de Guzzi Plepis A.M., da Conceição Amaro Martins V., dos Santos G.R., Pinto C.A.L., Pelegrine A.A., Teixeira M.L., Buchaim D.V., Nazari F.M. (2023). Collagen-chitosan-hydroxyapatite composite scaffolds for bone repair in ovariectomized rats. Sci. Rep..

[17] Pettian M.S., De Guzzi Plepis A.M., Da Conceição Amaro Martins V., Dos Santos G.R., Lopes Pinto C.A., Galdeano E.A., Alves Calegari A.R., De Moraes C.A., Da Cunha M.R. (2018). Use of an anionic collagen matrix made from bovine intestinal serosa for in vivo repair of cranial defects. PLoS ONE.

[18] Silva S.K., Plepis A.M.G., da Martins V.C.A., Horn M.M., Buchaim D.V., Buchaim R.L., Pelegrine A.A., Silva V.R., Kudo M.H.M., Fernandes J.F.R. (2022). Suitability of Chitosan Scaffolds with Carbon Nanotubes for Bone Defects Treated with Photobiomodulation. Int. J. Mol. Sci..

[19] De Oliveira E Silva M., Pelegrine A.A., Alves Pinheiro Da Silva A., Manhães Júnior L.R., De Mello E Oliveira R., Gaiba França S., Aloise A.C., Ferreira L.M. (2012). Xenograft enriched with autologous bone marrow in inlay reconstructions: A tomographic and histomorphometric study in rabbit calvaria. Int. J. Biomater..

[20] Liu S., Zhou C., Mou S., Li J., Zhou M., Zeng Y., Luo C., Sun J., Wang Z., Xu W. (2019). Biocompatible graphene oxide–collagen composite aerogel for enhanced stiffness and in situ bone regeneration. Mater. Sci. Eng. C.

[21] Sorushanova A., Delgado L.M., Wu Z., Shologu N., Kshirsagar A., Raghunath R., Mullen A.M., Bayon Y., Pandit A., Raghunath M. (2019). The Collagen Suprafamily: From Biosynthesis to Advanced Biomaterial Development. Adv. Mater..

[22] Puzio I., Graboś D., Bieńko M., Radzki R.P., Nowakiewicz A., Kosior-Korzecka U. (2021). Camelina oil supplementation improves bone parameters in ovariectomized rats. Animals.

[23] Orhan C., Sahin E., Er B., Tuzcu M., Lopes A.P., Sahin N., Juturu V., Sahin K. (2021). Effects of exercise combined with undenatured type ii collagen on endurance capacity, antioxidant status, muscle lipogenic genes and e3 ubiquitin ligases in rats. Animals.

[24] Chattopadhyay S., Raines R.T. (2014). Review collagen-based biomaterials for wound healing. Biopolymers.

[25] Sionkowska A., Wisniewski M., Skopinska J., Kennedy C.J., Wess T.J. (2004). Molecular interactions in collagen and chitosan blends. Biomaterials.

[26] Kalogera S., Jansen M., Bay Jensen A.-C., Frederiksen P., Karsdal M., Lafeber F., Mastbergen S., Thudium C. (2023). Relevance of Biomarkers in Serum vs Synovial Fluid in Patients with Knee Osteoarthritis. Int. J. Mol. Sci..

[27] De Oliveira V.M., Carneiro M., Pajeú T., Días C., de Souza R., Figueiredo A. (2017). Collagen: General characteristics and production of bioactive peptides—A review with emphasis on byproducts of fish. Acta Pesca Y Recur. Acuáticos.

[28] Trindade D., Cordeiro R., José H.C., Ângelo D.F., Alves N., Moura C. (2021). Biological treatments for temporomandibular joint disc disorders: Strategies in tissue engineering. Biomolecules.

[29] Young R.G., Butler D.L., Weber W., Caplan A.I., Gordon S.L., Fink D.J. (1998). Use of mesenchymal stem cells in a collagen matrix for achilles tendon repair. J. Orthop. Res..

[30] Grover C.N., Cameron R.E., Best S.M. (2012). Investigating the morphological, mechanical and degradation properties of scaffolds comprising collagen, gelatin and elastin for use in soft tissue engineering. J. Mech. Behav. Biomed. Mater..

[31] Rezvani Ghomi E., Nourbakhsh N., Akbari Kenari M., Zare M., Ramakrishna S. (2021). Collagen-based biomaterials for biomedical applications. J. Biomed. Mater. Res.—Part B Appl. Biomater..

[32] Moreira P.L., An Y.H., Rodrigues Santos A., Candelária Genari S. (2004). In vitro analysis of anionic collagen scaffolds for bone repair. J. Biomed. Mater. Res.—Part B Appl. Biomater..

[33] Maisani M., Pezzoli D., Chassande O., Mantovani D. (2017). Cellularizing hydrogel-based scaffolds to repair bone tissue: How to create a physiologically relevant micro-environment?. J. Tissue Eng..

[34] Rico-Llanos G.A., Borrego-González S., Moncayo-Donoso M., Becerra J., Visser R. (2021). Collagen type i biomaterials as scaffolds for bone tissue engineering. Polymers.

[35] Yang X., Han G., Pang X., Fan M. (2020). Chitosan/collagen scaffold containing bone morphogenetic protein-7 DNA supports dental pulp stem cell differentiation in vitro and in vivo. J. Biomed. Mater. Res.—Part A.

[36] Tatullo M., Codispoti B., Paduano F., Nuzzolese M., Makeeva I. (2019). Strategic tools in regenerative and translational dentistry. Int. J. Mol. Sci..

[37] Lee Y.C., Chan Y.H., Hsieh S.C., Lew W.Z., Feng S.W. (2019). Comparing the osteogenic potentials and bone regeneration capacities of bone marrow and dental pulp mesenchymal stem cells in a rabbit calvarial bone defect model. Int. J. Mol. Sci..

[38] Gendviliene I., Simoliunas E., Alksne M., Dibart S., Jasiuniene E., Cicenas V., Jacobs R., Bukelskiene V., Rutkunas V. (2021). Effect of extracellular matri x and dental pulp stem cells on bone regenerati on WI th 3D pri nted PLA/HA composi te scaffolds. Eur. Cells Mater..

[39] Morsczeck C., Reichert T.E. (2018). Dental stem cells in tooth regeneration and repair in the future. Expert Opin. Biol. Ther..

[40] Telles P.D., de Machado M.A.A.M., Sakai V.T., Nör J.E. (2011). Pulp tissue from primary teeth: New source of stem cells. J. Appl. Oral Sci..

[41] Shi X., Mao J., Liu Y. (2020). Pulp stem cells derived from human permanent and deciduous teeth: Biological characteristics and therapeutic applications. Stem Cells Transl. Med..

[42] Gross T., Dieterle M.P., Vach K., Altenburger M.J., Hellwig E., Proksch S. (2023). Biomechanical Modulation of Dental Pulp Stem Cell (DPSC) Properties for Soft Tissue Engineering. Bioengineering.

[43] Anitua E., Troya M., Zalduendo M. (2018). Progress in the use of dental pulp stem cells in regenerative medicine. Cytotherapy.

[44] Klinger Y., Leite D.C., Oliveira D.J., Quelemes P.V., Neto A., Ernanda C., De Carvalho S., Waleska H., Rodrigues S., Mu M. (2023). Novel Scaffold Based on Chitosan Hydrogels/Phthalated Cashew Gum for Supporting Human Dental Pulp Stem Cells. Pharmaceuticals.

[45] Fomby P., Cherlin A.J., Hadjizadeh A., Doillon C.J., Sueblinvong V., Weiss D.J., Bates J.H.T., Gilbert T., Liles W.C., Lutzko C. (2010). Stem cells and cell therapies in lung biology and diseases: Conference report. Ann. Am. Thorac. Soc..

[46] Fahimipour F., Dashtimoghadam E., Mahdi Hasani-Sadrabadi M., Vargas J., Vashaee D., Lobner D.C., Jafarzadeh Kashi T.S., Ghasemzadeh B., Tayebi L. (2019). Enhancing cell seeding and osteogenesis of MSCs on 3D printed scaffolds through injectable BMP2 immobilized ECM-Mimetic gel. Dent. Mater..

[47] Massimino L.C. (2020). Scaffolds de Biopolímeros e Resina de Jatobá Para Utilização em Engenharia Tecidual.

[48] Horn M.M., Martins V.C.A., de Guzzi Plepis A.M. (2015). Influence of collagen addition on the thermal and morphological properties of chitosan/xanthan hydrogels. Int. J. Biol. Macromol..

[49] Nwe N., Furuike T., Tamura H. (2009). The mechanical and biological properties of chitosan scaffolds for tissue regeneration templates are significantly enhanced by chitosan from Gongronella butleri. Materials.

[50] Zahedi S., Legrand R., Brunel G., Albert A., Dewé W., Coumans B., Bernard J. (1998). Evaluation of a Diphenylphosphorylazide-Crosslinked Collagen Membrane for Guided Bone Regeneration in Mandibular Defects in Rats. J. Periodontol..

[51] Bet M.R., Goissis G., Lacerda C.A. (2001). Characterization of polyanionic collagen prepared by selective hydrolysis of asparagine and glutamine carboxyamide side chains. Biomacromolecules.

[52] Goissis G., Suzigan S., Parreira D.R., Maniglia J.V., Braile D.M., Raymundo S. (2000). Preparation and characterization of collagen-elastin matrices from blood vessels intended as small diameter vascular grafts. Artif. Organs.

[53] Buchaim R.L., Goissis G., Andreo J.C., Roque D.D., Roque J.S., Buchaim D.V., de Rodrigues A.C. (2007). Biocompatibility of anionic collagen matrices and its influence on the orientation of cellular growth. Braz. Dent. Sci..

[54] Massimino L.C., da Conceição Amaro Martins V., Vulcani V.A.S., de Oliveira É.L., Andreeta M.B., Bonagamba T.J., Klingbeil M.F.G., Mathor M.B., de Guzzi Plepis A.M. (2020). Use of collagen and auricular cartilage in bioengineering: Scaffolds for tissue regeneration. Cell Tissue Bank.

[55] Milan E.P., Rodrigues M.Á.V., Martins V.C.A., Plepis A.M.G., Fuhrmann-Lieker T., Horn M.M. (2021). Mineralization of phosphorylated fish skin collagen/mangosteen scaffolds as potential materials for bone tissue regeneration. Molecules.

[56] Zam Z.Z., Muin F., Fataruba A. (2021). Identification of chitosan beads from coconut crab patani variety using Fourier Transform Infrared Spectroscopy (FTIR). J. Phys. Conf. Ser..

[57] Battafarano G., Rossi M., De Martino V., Marampon F., Borro L., Secinaro A., Fattore A. (2021). Del Strategies for bone regeneration: From graft to tissue engineering. Int. J. Mol. Sci..

[58] Zhang K., Fan Y., Dunne N., Li X. (2018). Effect of microporosity on scaffolds for bone tissue engineering. Regen. Biomater..

[59] Horn M.M., Martins V.C.A., de Guzzi Plepis A.M. (2009). Interaction of anionic collagen with chitosan: Effect on thermal and morphological characteristics. Carbohydr. Polym..

[60] Karageorgiou V., Kaplan D. (2005). Porosity of 3D biomaterial scaffolds and osteogenesis. Biomaterials.

[61] Iacob A.T., Drăgan M., Gheţu N., Pieptu D., Vasile C., Buron F., Routier S., Giusca S.E., Caruntu I.D., Profire L. (2018). Preparation, characterization and wound healing effects of new membranes based on chitosan, hyaluronic acid and arginine derivatives. Polymers.

[62] Grabska-Zielińska S., Sionkowska A., Coelho C.C., Monteiro F.J. (2020). Silk fibroin/collagen/chitosan scaffolds cross-linked by a glyoxal solution as biomaterials toward bone tissue regeneration. Materials.

[63] Arun A., Malrautu P., Laha A., Ramakrishna S. (2021). Gelatin Nanofibers in Drug Delivery Systems and Tissue Engineering. Eng. Sci..

[64] Liu P., Zhang Y., Ma Y., Tan S., Ren B., Liu S., Dai H., Xu Z. (2022). Application of dental pulp stem cells in oral maxillofacial tissue engineering. Int. J. Med. Sci..

[65] de Moraes R., de Guzzi Plepis A.M., da Conceição Amaro Martins V., Duarte M.A.H., Alcalde M.P., Buchaim R.L., Pomini K.T., Machado E.G., de Azevedo E Sousa Munhoz M., Cunha F.B. (2019). Suitability of the use of an elastin matrix combined with bone morphogenetic protein for the repair of cranial defects. Am. J. Transl. Res..

[66] Della Coletta B.B., Jacob T.B., de Moreira L.A.C., Pomini K.T., Buchaim D.V., Eleutério R.G., de Pereira E.S.B.M., Roque D.D., de Rosso M.P.O., Shindo J.V.T.C. (2021). Photobiomodulation Therapy on the Guided Bone Regeneration Process in Defects Filled by Biphasic Calcium Phosphate Associated with Fibrin Biopolymer. Molecules.

[67] Nie X., Wang D.A. (2018). Decellularized orthopaedic tissue-engineered grafts: Biomaterial scaffolds synthesised by therapeutic cells. Biomater. Sci..

[68] Wollmann L., Suss P., Mendonça J., Luzia C., Schittini A., da Rosa G.W.X., Costa F., Tuon F.F. (2019). Characterization of decellularized human pericardium for tissue engineering and regenerative medicine applications. Arq. Bras. Cardiol..

[69] Hussein K.H., Park K.M., Kang K.S., Woo H.M. (2016). Biocompatibility evaluation of tissue-engineered decellularized scaffolds for biomedical application. Mater. Sci. Eng. C.

[70] Latimer J.M., Maekawa S., Yao Y., Wu D.T., Chen M., Giannobile W.V. (2021). Regenerative Medicine Technologies to Treat Dental, Oral, and Craniofacial Defects. Front. Bioeng. Biotechnol..

[71] Ahmadi F., Oveisi Z., Samani M., Amoozgar Z. (2015). Chitosan based hydrogels: Characteristics and pharmaceutical applications. Res. Pharm. Sci..

[72] Rodríguez-Vázquez M., Vega-Ruiz B., Ramos-Zúñiga R., Saldaña-Koppel D.A., Quiñones-Olvera L.F. (2015). Chitosan and Its Potential Use as a Scaffold for Tissue Engineering in Regenerative Medicine. Biomed Res. Int..

[73] Nogueira D.M.B., de Figadoli A.L.F., Alcantara P.L., Pomini K.T., Santos German I.J., Reis C.H.B., Rosa Júnior G.M., de Rosso M.P.O., da Santos P.S.S., Zangrando M.S.R. (2022). Biological Behavior of Xenogenic Scaffolds in Alcohol-Induced Rats: Histomorphometric and Picrosirius Red Staining Analysis. Polymers.

[74] Zdilla M.J., Pancake J.P., Russell M.L., Koons A.W. (2022). Ontogeny of the human fetal, neonatal, and infantile basioccipital bone: Traditional and extended eigenshape geometric morphometric analysis. Anat. Rec..

[75] Jégoux F., Goyenvalle E., Cognet R., Malard O., Moreau F., Daculsi G., Aguado E. (2010). Mandibular segmental defect regenerated with macroporous biphasic calcium phosphate, collagen membrane, and bone marrow graft in dogs. Arch. Otolaryngol.—Head Neck Surg..

[76] Soares D.G., Rosseto H.L., Basso F.G., Scheffel D.S., Hebling J., Costa C.A.D.S. (2016). Chitosan-Collagen Biomembrane Embedded With Calcium-Aluminate Enhances Dentinogenic Potential Of Pulp Cells. Braz. Oral Res..

[77] Soares D.G., Rosseto H.L., Scheffel D.S., Basso F.G., Huck C., Hebling J., de Souza Costa C.A. (2017). Odontogenic differentiation potential of human dental pulp cells cultured on a calcium-aluminate enriched chitosan-collagen scaffold. Clin. Oral Investig..

[78] Chamieh F., Collignon A.M., Coyac B.R., Lesieur J., Ribes S., Sadoine J., Llorens A., Nicoletti A., Letourneur D., Colombier M.L. (2016). Accelerated craniofacial bone regeneration through dense collagen gel scaffolds seeded with dental pulp stem cells. Sci. Rep..

[79] Van Leeuwen A.C., Huddleston Slater J.J.R., Gielkens P.F.M., De Jong J.R., Grijpma D.W., Bos R.R.M. (2012). Guided bone regeneration in rat mandibular defects using resorbable poly(trimethylene carbonate) barrier membranes. Acta Biomater..

[80] Hoogeveen E.J., Gielkens P.F.M., Schortinghuis J., Ruben J.L., Huysmans M.C.D.N.J.M., Stegenga B. (2009). Vivosorb® as a barrier membrane in rat mandibular defects. An evaluation with transversal microradiography. Int. J. Oral Maxillofac. Surg..

[81] de Azevedo e Sousa Munhoz M., Pomini K.T., de Guzzi Plepis A.M., da Conceição Amaro Martins V., Machado E.G., de Moraes R., Cunha F.B., Santos A.R., Cardoso G.B.C., Duarte M.A.H. (2020). Elastin-derived scaffolding associated or not with bone morphogenetic protein (BMP) or hydroxyapatite (HA) in the repair process of metaphyseal bone defects. PLoS ONE.

[82] Iatecola A., Longhitano G.A., Antunes L.H.M., Jardini A.L., de Miguel E.C., Béreš M., Lambert C.S., Andrade T.N., Buchaim R.L., Buchaim D.V. (2021). Osseointegration improvement of co-cr-mo alloy produced by additive manufacturing. Pharmaceutics.

[83] MacArthur Clark J. (2018). The 3Rs in research: A contemporary approach to replacement, reduction and refinement. Br. J. Nutr..

[84] Camilli J.A., Da Cunha M.R., Bertran C.A., Kawachi E.Y. (2004). Subperiosteal hydroxyapatite implants in rats submitted to ethanol ingestion. Arch. Oral Biol..

[85] Yao S., Du Z., Xiao L., Yan F., Ivanovski S., Xiao Y. (2022). Morphometric Changes of Osteocyte Lacunar in Diabetic Pig Mandibular Cancellous Bone. Biomolecules.

[86] Mesh P.L., Paniculate A., Bone C., Kinoshita Y., Ph D., Kobayashi M. (1996). Functional Reconstruction of the Jaw Bones Using. Tissue Eng..

[87] Phutane P., Telange D., Agrawal S., Gunde M., Kotkar K., Pethe A. (2023). Biofunctionalization and Applications of Polymeric Nanofibers in Tissue Engineering and Regenerative Medicine. Polymers.

[88] Nyman S., Gottlow J., Lindhe J., Karring T., Wennstrom J. (1987). New attachment formation by guided tissue regeneration. J. Periodontal Res..

[89] Wessing B., Lettner S., Zechner W. (2018). Guided Bone Regeneration with Collagen Membranes and Particulate Graft Materials: A Systematic Review and Meta-Analysis. Int. J. Oral Maxillofac. Implants.

[90] Bružauskaitė I., Bironaitė D., Bagdonas E., Bernotienė E. (2016). Scaffolds and cells for tissue regeneration: Different scaffold pore sizes—Different cell effects. Cytotechnology.

