# Supplementary figures and images for: In Vivo Evaluation of Collagen and Chitosan Scaffold, Associated or Not with Stem Cells, in Bone Repair
Source: J Funct Biomater. 2023 Jul 8;14(7):357. doi: 10.3390/jfb14070357 (PMC10381363; doi:10.3390/jfb14070357)

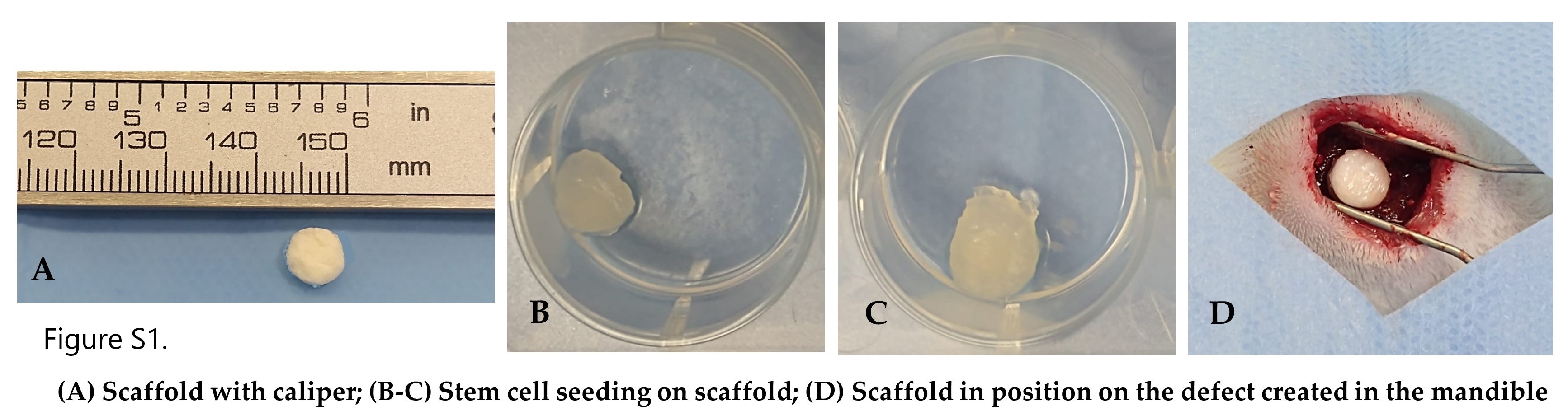

Supplement: Supplementary file 1 [file jfb-14-00357-s001.zip › Figure S1.jpg]

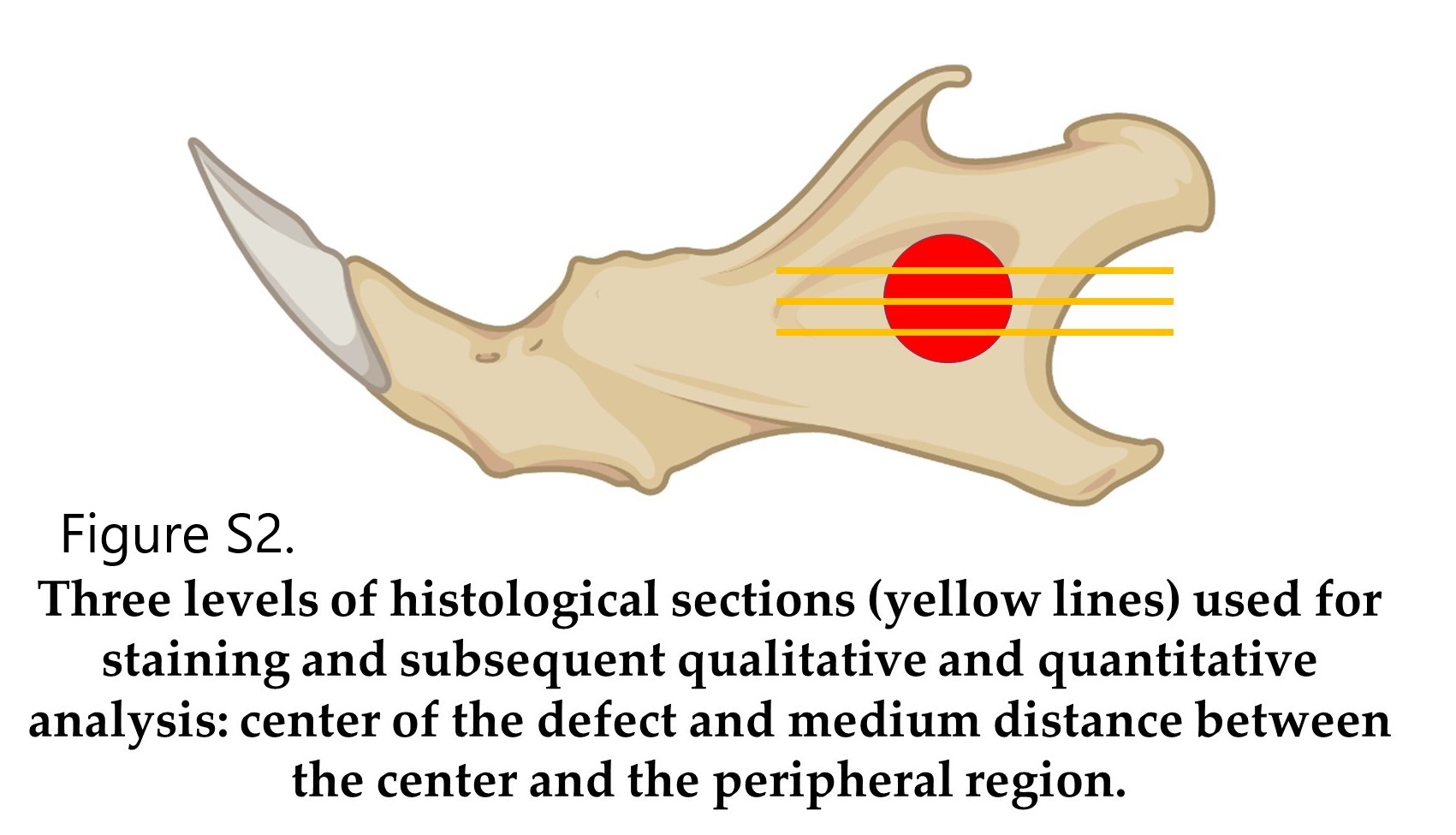

Supplement: Supplementary file 1 [file jfb-14-00357-s001.zip › Figure S2.jpg]
